# Prevalence of cardiac dysfunction and longitudinal changes in cardiac function after breast cancer treatment with chemotherapy with/without radiation therapy compared with controls

**DOI:** 10.1016/j.breast.2026.104781

**Published:** 2026-04-04

**Authors:** L.T. van der Wal, S.W.M.C. Maass, M.R. de Boer, M.H. Blanker, A.P.G. Crijns, J.A. Gietema, P. van der Meer, G.H. de Bock, D. Brandenbarg

**Affiliations:** a University of Groningen, University Medical Center Groningen, Department of Primary and Long-Term Care, Cure and Care in the Community Context (FOUR-C) research program, Hanzeplein 1, HPC: FA21, PO Box 196, Groningen, 9700 RB, the Netherlands; b University of Groningen, University Medical Center Groningen (UMCG), Department of Radiation Oncology, Hanzeplein 1, P.O. Box 30.001, 9700 RB, Groningen, the Netherlands; c University of Groningen, University Medical Center Groningen (UMCG), Department of Medical Oncology, Hanzeplein 1, PO Box 30.001, Groningen, 9700 RB, the Netherlands; d University of Groningen, University Medical Center Groningen (UMCG), Department of Cardiology, Hanzeplein 1, PO Box 30.001, Groningen, 9700 RB, the Netherlands; e University of Groningen, University Medical Center Groningen (UMCG), Department of Epidemiology, Hanzeplein 1, HPC: FA40, PO Box 30.001, Groningen, 9700 RB, the Netherlands

**Keywords:** Breast cancer, Survivors, Echocardiography, Left ventricular systolic dysfunction, Long-term adverse effects, General practitioner

## Abstract

**Introduction:**

Breast cancer survivors are known to have a higher prevalence of mild systolic cardiac dysfunction compared to the general population, due to the effects of chemotherapy with/without radiation therapy. However, the long-term prevalence of cardiac dysfunction and changes in (dys)function over time, remain unclear.

**Materials and methods:**

We added a follow-up measurement to a matched-cohort study. Women were recruited from general practices, and echocardiographic data and medical records from general practices were collected for analysis. The primary outcome was the prevalence of mild left ventricular systolic dysfunction, defined as a left ventricular ejection fraction (LVEF) <54%. Secondary outcomes were diastolic dysfunction, right ventricular dysfunction and longitudinal changes of LVEF over time.

**Results:**

Of the 700 women enrolled in the initial study (T1), 336 (48%) were included in the follow-up (T2). At T2, the median age was 70 years (IQR 65–75) for breast cancer survivors, with a median follow-up time of 19 years (IQR 17–22) after diagnosis. The prevalence of mild left ventricular systolic dysfunction was 10.4% among breast cancer survivors, versus 3.5% of controls (OR 3.1, 95% CI 1.2–8.1). Mixed-effects modeling showed that LVEF slightly improved over time across groups, without interactions between timepoint and group.

**Conclusion and discussion:**

Long-term breast cancer survivors who received chemotherapy with/without radiation therapy are at increased risk of mild left ventricular systolic dysfunction compared to controls. Despite this elevated risk, left ventricular function slightly improved for all women over time and remained clinically irrelevant.

## Highlights

• Breast cancer survivors show higher prevalence of mild LV systolic dysfunction.
• Left ventricular systolic function does not deteriorate over time.
• Routine long-term cardiac monitoring may be unnecessary.

## 1 Introduction

Female breast cancer, with nearly 2.3 million new cases in 2020, is the most diagnosed cancer globally. In high income countries, its incidence has increased due to enhanced detection through screening and is expected to continue to rise, driven by population ageing and lifestyle factors [1]. Importantly, survival has also improved substantially in these regions, mainly because of better staging and significant advances in therapeutic strategies [2]. As a result, five-year survival rates are approaching 100% and 80% for women with stage I and stage III breast cancer, respectively [3]. These trends reflect an ever growing number of breast cancer survivors, and underscore the need to address the long-term effects of cancer and its therapy [1].

Recent literature shows that breast cancer therapy, typically comprising chemotherapy, radiation therapy, and sometimes targeted agents such as trastuzumab, can affect cardiac function even a decade after treatment [[4], [5], [6]]. These therapies are associated with cardiotoxicity, characterized by an increased risk of left ventricular dysfunction, especially at high cumulative doses or when combined [7]. Additional factors such as multiple cardiovascular risk factors, older age and pre-existing impaired cardiac function further increase the likelihood of cardiotoxicity [5]. Cardiac dysfunction represents an early stage on the pathway to heart failure [8]. Because of its gradual onset and nonspecific symptoms such as fatigue, cardiac dysfunction often remains unrecognized, despite being amenable to early intervention. Once overt clinical symptoms appear, heart failure may be irreversible [9,10].

During and shortly after cancer treatment, patients who received chemotherapy are closely monitored in hospital-based cardio-oncology programs. Once this care ends, routine cardiac surveillance is only continued in case of existing cardiac dysfunction [10]. Consequently, many breast cancer survivors only seek medical attention of their general practitioner after overt clinical symptoms of cardiac dysfunction have developed. According to the European Society of Cardiology (ESC) guideline, follow-up surveillance is advised for breast cancer survivors treated with high dose anthracyclines or those who developed cardiotoxicity during chemotherapy [10]. Studies on long-term cardiac outcomes are scare. Most studies to date had a follow-up period of no more than 10 years after diagnosis, were conducted in hospital-based settings, lacked a control population, or were performed in a population with symptoms of cardiac dysfunction which may have led to an overestimation of the true prevalence of cardiac dysfunction. The only study performed in primary care, was our initial study showing that breast cancer survivors have an increased risk of mild systolic cardiac dysfunction compared with matched controls on average 10 years after diagnosis [11]. How this dysfunction changes over the long-term remains uncertain. To address this gap, a follow-up measurement was added, to assess cardiac dysfunction prevalence and trajectory of cardiac function in the same populations.

## 2 Methods

### 2.1 Study design

A longitudinal matched-cohort study was created by adding a follow-up measurement to the initial study [11]. In this initial study, survivors of patients with early breast cancer (stage I-III) treated with chemo and/or radiation therapy were matched to women of the same age (±1 year) and general practitioner (GP), but without any history of cancer or cancer treatment. In our present follow-up study, data from the first assessment (T1) was combined with new data (T2). The medical ethics committee of the University Medical Center Groningen (UMCG) approved this study, all participants gave written informed consent, and a protocol was registered publicly (NL81110.042.22, ClinicalTrials.gov ID NCT05851053).

### 2.2 Participants

The participants from the initial study were re-invited to participate in a second follow-up assessment. The GP assessed which women were still alive and registered in their GP practice. Eligible women, who were able to visit the hospital, as assessed by their treating GP, were invited by their GP by letter.

### 2.3 Study assessment and procedures

Eligible women willing to participate returned a signed informed consent form and were scheduled for echocardiography. For individuals who declined participation, the reason for non-participation was documented.

The initial dataset (T1) included data on cardiovascular (CV) risk factors and cardiovascular disease (CVD) extracted from general practice records using the International Classification of Primary Care codes (ICPC) [12]. For breast cancer survivors, additional treatment information was obtained from hospital charts, including chemotherapy regimens, cumulative anthracycline doses, antihormonal therapy, and radiation therapy site [11]. At T1, all participants underwent standardized echocardiography.

For the current follow-up study (T2), data was collected on CV risk factors, presence of CVD, and cardiac function using the same standardized procedures. Data on CV risk factors and CVD were collected from the electronic GP records using ICPC codes, including corresponding dates of diagnosis. Cardiovascular risk factors were lipid metabolism disorders (T93), hypertension (K86) or diabetes mellitus (T90), diagnosed by the GP. Heart failure (K77), ischemic cardiovascular disease (K74, K75, K76, K89 and K90), atrial fibrillation (K78), other cardiac diseases (K79, K83, K84) were defined as CVD. Additionally, information about recurrence, contralateral breast cancer, metastases and new types of cancer for women participating in this follow-up study was collected from the electronic GP records.

Echocardiography was performed, using a Vivid E95 ultrasound machine (GE, Horten, Norway), by experienced personnel from the cardiology department of the UMCG. All echocardiographic assessments were performed by personnel who were blinded to participants’ breast cancer history. Echocardiography was performed at the same center as the initial assessment, according to the guidelines of the European Association of Echocardiography. A prespecified imaging protocol was applied and images were digitally stored [13,14]. Collected data were stored into a separate, anonymous, password-protected database (Research Electronic Data Capture: RedCap).

### 2.4 Primary outcome

The primary outcome was the prevalence of left ventricular systolic dysfunction, defined as a left ventricular ejection fraction (LVEF) <54% according to the European Association for Cardio Vascular Imaging (EACVI) [15]. This was measured by the biplane method of disks summation (the modified Simpsons's rule) [13]. In cases of insufficient image quality, an estimation of LVEF was provided by the ultrasound technician through eyeballing.

### 2.5 Secondary outcomes

Secondary outcomes included LVEF <50% and <45%, global longitudinal strain >−16% (GLS), diastolic dysfunction and course of LVEF. Diastolic dysfunction was defined according to the 2016 EACVI guidelines, with a distinction made between dysfunction occurring in the context of preserved versus impaired LVEF [14]. In addition, diastolic dysfunction was defined by a left atrial volume index (LAVI) greater than 34 ml/m^2^ and an E/e’ ratio exceeding 14. Right ventricular systolic dysfunction was defined by a decreased tricuspid annular plane systolic excursion (TAPSE) (<17 mm) and having decreased S' (<9.5 cm/s) [14]. The trajectory of LVEF was evaluated by performing quantitative assessments at two distinct time points (T1 and T2).

### 2.6 Statistical analysis

Age was derived from the recorded year and month of birth. Details about breast cancer treatment and CV risk factors and CVD at the time of breast cancer diagnosis were obtained, and compared between included women and women lost to follow-up. Median and interquartile ranges were given for continuous variables. Frequencies were calculated for categorical variables.

Our primary outcome, the prevalence of cardiac dysfunction in breast cancer survivors at follow-up measurement, was compared to controls using univariate logistic regression by estimating odds ratio's (OR) and their 95% confidence intervals (95% CI). Additionally, the prevalence on cardiac dysfunction was compared for women treated with chemotherapy with or without radiation therapy and controls, and for women treated with radiation therapy only and controls. Statistical analyses were performed in R (version 4.4.1), employing the packages dplyr for data manipulation, lubridate for date-time handling, epitools for epidemiological computations, and tidyr for data restructuring.

Our sample size calculation was based on the expected difference in the prevalence of left ventricular systolic dysfunction between breast cancer survivors and controls {Boerman, 2017 #21}. We hypothesized a prevalence of left ventricular systolic dysfunction of 20% in breast cancer survivors and 10% in controls. Using these estimates with a beta of 0.80 and alpha of 0.05, we calculated a required sample size of 197 participants per group.

The trajectory of systolic function over time was analyzed using a linear mixed effects model, to account for the within subject correlations between LVEF measurements. Left ventricular ejection fraction served as the outcome variable. Treatment group, timepoint of assessment (I vs. II), and their interaction were included as fixed effects, with a random intercept at the individual level. The model was fitted using the lme4 package in R. Estimated marginal means were obtained through the emmeans package and statistical inference for fixed effects was conducted using the pbkrtest package.

## 3 Results

**Fig. 1:**
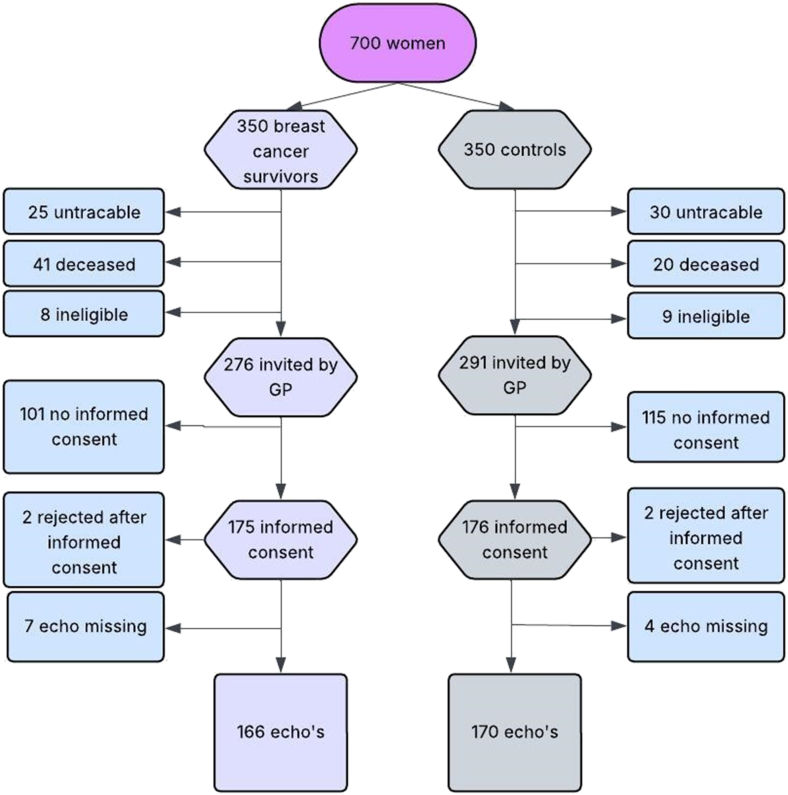
Flow diagram for the selection of breast cancer survivors and matched controls.

Of the 700 women included in the initial study, 55 were untraceable and 61 had died. Among the breast cancer survivors, 41 deaths were recorded (Fig. 1). In two breast cancer survivors, both treated with radiation therapy only, heart failure was identified as the cause of death. In the control group heart failure was reported once as the cause of death. Of the remaining women, 567 were deemed eligible and invited by their GP, of whom 351 (61.9%) gave informed consent. Four women withdrew after providing informed consent, and eleven women did not show up at their appointments, leaving echocardiographic assessments for 166 breast cancer survivors and 170 controls (Fig. 1).

### 3.1 Participant characteristics

The median age was 69.9 years (IQR 64.5-75.3) for the breast cancer survivors and 70.8 years (IQR 64.1-75.6) for controls, and median follow-up time was 19.1 years (IQR 16.6−22.4) after diagnosis (Table 1). For women treated with radiation therapy only, the median age was 73.0 years (IQR 68.1−76.8), and for women treated with chemotherapy with or without radiation therapy 67.2 years (IQR 61.3−71.8). Among breast cancer survivors, over half received chemotherapy (54.2%), of which the majority received anthracycline based chemotherapy, while 80.7% were treated with radiation therapy. The median cumulative anthracycline dose was 252.9 mg/m^2^ (IQR 236.0−299.6). Cardiovascular risk factors and CVD, as documented in the GP records, were prevalent in 7.8% and 1.2% of breast cancer survivors, respectively. One woman in the control group died after undergoing her echocardiographic assessment and before primary care record review was conducted (Table 1). For women lost to follow-up, CV risk factors were prevalent in 13.6% of breast cancer survivors and 13.3% of controls and CVD was prevalent in 2.2% of breast cancer survivors and 2.8% of controls. A total of 22.3% of breast cancer survivors died during follow-up, compared with 11.1% of women in the control group (Table A.1, Appendix).

**Table 1:** Baseline characteristics at time of breast cancer diagnosis for breast cancer survivors and controls for women with a follow-up measurement.

	Breast cancer survivors (N = 166)	Controls (N = 170)
Age breast cancer diagnosis, years, median (IQR)	50.0 (43.8 - 55.1)	51.0 (43.8 - 56.0)^a^
Year of diagnosis, year, median (IQR)	2004.5 (2001.3 - 2006.8)	2005.4 (2000.8 - 2007.4)^a^
Age at T2, years, median (IQR)	69.9 (64.5 - 75.3)	70.8 (64.1 - 75.6)
Follow-up duration from diagnosis to T2, years, median (IQR)	19.1 (16.6 - 22.4)	18.5 (16.3 - 22.8)^a^

	N (%)	N (%)

Chemotherapy	90 (54.2)	-
Antracycline based chemotherapy	73 (44.0)	-
Cumulative anthracycline dose; mg/m2, median (IQR)^b^	252.9 (236.0 - 299.6)	-
Radiation therapy	134 (80.7)	-
Left sided radiation therapy	72 (43.4)	-
Hormonal therapy	75 (45.2)	-
Trastuzumab	9 (5.4)	-

**Cardiovascular risk factor**^c^	13 (7.8)	18 (10.6)
**Cardiovascular disease**^d^	2 (1.2)	2 (1.2)
**Deceased**	0 (0)	1 (0.6)

T2: Assessment 2.

a Age at breast cancer diagnosis and follow-up, year of diagnosis, and follow-up duration were calculated based on the matched breast cancer survivor.

b Doxorubicin isotoxic dose, information available for 55 patients. Matching was performed on general practice and age, ensuring comparability between cases and controls.

c Lipid metabolism disorders, hypertension or diabetes mellitus.

d Heart failure, ischemic cardiovascular disease, atrial fibrillation, other cardiac diseases.

### 3.2 Primary outcome

Left ventricular ejection fraction <54% was found in 10.2% of the breast cancer survivors, versus 3.5% in controls (OR 3.1, 95% CI 1.2−8.1) (Table 2). From the 17 breast cancer survivors with LVEF <54%, 11 were treated with chemotherapy with or without radiation therapy and six were treated with radiation therapy only (Table A.2, Appendix). At T1, LVEF <54% was found in 16.3% breast cancer survivors versus 6.5% in controls (OR 2.9, 95% CI 1.4−6.0) (Table A.3, Appendix).

**Table 2:** The prevalence of left ventricular dysfunction, diastolic dysfunction and right ventricular dysfunction with a follow-up measurement and the comparison between breast cancer survivors and controls.

	Breast cancer survivors N = 166 (%)	Controls N = 170 (%)	OR (95% CI)
**Left ventricular dysfunction**			
LVEF <54%^a^	17 (10.2)	6 (3.5)	3.1 (1.2 - 8.1)
LVEF <50%^a^	10 (6.0)	5 (2.9)	2.1 (0.7 - 6.3)
LVEF <45%^a^	1 (0.6)	0 (0.0)	-
GLS >−16%	36 (28.3)	26 (18.8)	1.7 (1.0 - 3.0)
**Diastolic dysfunction**			
Diastolic dysfunction guideline 2016	2 (1.4)	2 (1.3)	1.1 (0.2 - 7.8)
Increased LAVI (>34 ml/m^2^)	34 (21.0)	27 (16.1)	1.4 (0.8 - 2.4)
Increased E/e'ratio (>14)	1 (0.6)	2 (1.2)	0.5 (0.0 - 5.9)
**Right ventricular dysfunction**			
Decreased TAPSE (<17 mm)	1 (0.6)	3 (1.8)	0.3 (0.0 - 3.3)
Decreased S' (<9.5 cm/s)	2 (1.3)	1 (0.6)	2.1 (0.2 - 23.3)

GLS % (median, IQR)	−17.8 (−19.4 to −15.8)	−18.5 (−19.8 to −16.7)	
E/e' ratio (median, IQR)	6.9 (5.9 - 7.9)	6.8 (5.7 - 8.0)	
LAVI >34 ml/m^2^ (median, IQR)	25.3 (21.8 - 32.8)	26.7 (23.6 - 31.0)	

LVEF: Left ventricular ejection fraction. GLS: Global longitudinal strain. LAVI: Left atrial volume index. TAPSE: tricuspid annular plane systolic excursion. OR: Odds ratio. CI: Confidence interval. BC: Breast cancer.

LVEF available for 166 BC survivors and 170 controls, GLS available for 127 BC survivors and 138 controls, Diastolic disfunction available for 146 BC survivors and 159 controls, LAVI available for 162 BC survivors and 168 controls, E/e’ ratio available for 154 BC survivors and 164 controls, TAPSE available for 166 BC survivors and 169 controls, S′ for 156 BC survivors and 162 controls.

a LVEF through eyeballing for 23 BC survivors and 15 controls.

### 3.3 Secondary outcomes

Left ventricular ejection fraction <50% was found in 6.0% of breast cancer survivors, versus 2.9% in controls and LVEF <45% was found in one breast cancer survivor. In 38 women eyeballing was used to determine LVEF (23 BC survivors and 15 controls) (Table 2). Global longitudinal strain >−16% was found in 28.3% of breast cancer survivors versus 18.8% of controls. From the 36 breast cancer survivors with GLS >−16%, the majority (n =23) were treated with chemotherapy with or without radiation therapy (Table A.2, Appendix). Additionally, diastolic dysfunction according to the guideline of 2016 was found in 1.4% of the breast cancer survivors compared to 1.3% of controls and a decreased TAPSE was found in 0.6% of breast cancer survivors and 1.8% of controls. The median GLS in breast cancer survivors was −17.8% versus −18.5% in controls (Table 2). In breast cancer survivors treated with chemotherapy with or without radiation therapy, the median GLS was −17.2%, compared to −18.8% of their controls (Table A.2, Appendix). Among our participants, seven (7.8%) breast cancer survivors who received chemotherapy with or without radiation therapy had recurrence and 12 (15.8%) women treated with radiation therapy only had recurrence. Both treatment groups included two women who developed metastases. In the chemotherapy with or without radiation therapy group four (4.4%) women were diagnosed with contralateral breast cancer compared with five (6.6%) women in the radiation therapy only group. In the control cohort, four (2%) women were diagnosed with breast cancer during follow-up (data not shown).

According to the mixed-effects model, all women, irrespectively of treatment, slightly improved in LVEF (p =0.78 for group × timepoint interaction). Breast cancer survivors who received chemotherapy with or without radiation therapy exhibit the lowest estimated mean LVEF with values of 57.4% at T1 (95% CI 56.7–58.2), and 58.1% at T2 (95% CI 57.1–59.1). Breast cancer survivors treated with radiation therapy exhibited LVEF outcomes of 58.1% (95% CI 57.4–58.9) at T1 and 59.6% (95% CI 58.5–60.7) at T2. Both control groups consistently showed the highest estimated LVEF values across timepoints with LVEF values above 59% at T1 and above 60% at T2 (Table 3).

**Table 3:** Mixed effects model of LVEF (%) at T1 and T2 between breast cancer survivors treated with chemotherapy with or without radiation therapy, radiation therapy only, and their controls.

Predictor	LVEF (%) T1	95% Confidence Interval	LVEF (%) T2	95% Confidence Interval
Chemotherapy with or without radiation therapy	57.4	56.7 - 58.2	58.1	57.1 - 59.1
Controls chemotherapy with or without radiation therapy	59.3	58.6 - 60.1	60.7	59.6 - 61.7
Radiation therapy	58.1	57.4 - 58.9	59.6	58.5 - 60.7
Controls radiation therapy	59.1	58.4 - 59.9	60.4	59.4 - 61.4

T1: Assessment 1. T2: Assessment 2.

## 4 Discussion

In this cohort study of women recruited from primary care, we assessed the prevalence of cardiac dysfunction and trajectory in systolic function in breast cancer survivors, at a median of 19 years post diagnosis. Patients with early-stage breast cancer treated with adjuvant chemotherapy, radiation therapy or a combination of both exhibited a higher prevalence of mild systolic cardiac dysfunction (10.2%) than controls (3.5%). Longitudinal analyses indicated a small improvement in LVEF over 9 years in all women, irrespectively of breast cancer treatment.

### 4.1 Prevalence of left ventricular systolic dysfunction

The prevalence of 10.2% in our cohort is consistent with findings from other studies on left ventricular systolic dysfunction in breast cancer survivors with shorter follow-up. For instance, a cross-sectional study with a median follow-up period of 5 to 12 years reported that 10% of breast cancer survivors treated with anthracyclines had a LVEF <54%, compared with 4% among those who did not receive anthracyclines. The median cumulative anthracycline exposure in that study was 240 mg/m^2^, which closely aligns with the 252.9 mg/m^2^ observed in our cohort, further supporting the comparability of dose-related findings across studies [16]. Likewise, a study with unselected breast cancer survivors in routine follow-up, documented a prevalence of systolic dysfunction (defined as LVEF <55%) of 11.5% among doxorubicin-treated survivors. However, echocardiographic evaluation in that study was restricted to individuals with elevated cardiac biomarkers and median follow-up time was 6 years [17]. Another cohort study documented a prevalence of 10.4% in breast cancer survivors with a median of 6.6 years after cardiotoxic therapy initiation, using a LVEF cut-off of <50% [18]. Importantly, when applying this stricter threshold in our cohort, the prevalence was substantially lower (6.0%).

### 4.2 Prevalence of diastolic dysfunction and right ventricular systolic disfunction

Diastolic dysfunction was observed in 2% of breast cancer survivors and 2% of controls. The prevalence we found is notably lower compared to several previous studies among long-term breast cancer survivors. However, these differences largely reflect changes in the diagnostic criteria for diastolic dysfunction over time. Our initial assessment, conducted approximately 10 years after breast cancer diagnosis, and based on the 2009 guideline for determining diastolic dysfunction, showed a prevalence of 43.4% in breast cancer survivors versus 39.5% in controls [11,19]. In another study with a median follow-up of 7 years, diastolic dysfunction, defined as e' lateral or e' septal at 2.5% below the normal range for each age group, was observed in 39.4% of breast cancer survivors [20]. A key factor contributing to the divergence in reported prevalence is the definition and diagnostic criteria for diastolic dysfunction utilized across studies. A study on the impact of the 2016 ASE/EACVI guideline revealed that this guideline resulted in a much lower prevalence of diastolic dysfunction in the general population (1.4%) compared to the guideline of 2009 (38.1%) [14,19,21].

### 4.3 Course of left ventricular systolic function

Our longitudinal analysis suggests that left ventricular systolic function slightly improves over time in breast cancer survivors as well as in controls, albeit clinically not relevant. Scientific literature on the trajectory of cardiac function in breast cancer survivors versus controls is scarce. The study from Bostany et al. (2025), with a median follow-up time of 8.6 years, performed a longitudinal analysis of breast cancer survivors with cardiac dysfunction (defined as LVEF <50%) and those without cardiac dysfunction. They reported an annual decline LVEF of 0.29% among survivors with cardiac dysfunction, whereas survivors without cardiac dysfunction showed an annual increase of 0.25% [18]. Notably, that study did not include a control group, only 56 women had a 20-year follow-up measurement, and the proportion of women with pre-existing CV risk factors was more than twice as high compared to our cohort (28.2% with ≥1 risk factor).

### 4.4 Strengths and limitations

A key strength of our study lies in the inclusion of participants in primary care. Our initial dataset included non-symptomatic breast cancer survivors and GP and age matched controls. This design allows for the assessment whether non-symptomatic breast cancer survivors in primary care are at increased risk of developing mild left ventricular systolic dysfunction. When interpreting our results, some limitations should be considered. It is possible that attrition bias influenced our results, if women with more severe systolic dysfunction were less likely to participate in the current study. Of the 276 breast cancer survivors and 291 controls invited by their GP, 216 women did not agree to consent and participate in the study. It is possible that these participants experienced worse outcomes than those who did consent. The modest increase in LVEF may reflect true physiological recovery, but it could also be influenced by survivor bias. Because image quality was insufficient in some ultrasounds, the biplane Simpson method could not always be used. In those cases, LVEF was visually estimated, which is more operator-dependent and may introduce additional variability. This heterogeneity could have affected classification around the 54% cut-off, potentially leading to some misclassification. Although the number of visually estimated measurements was small, a degree of measurement bias cannot be excluded. Despite the fact that only two women developed metastatic disease during follow-up, it is possible that additional systemic treatments associated with metastatic disease may have adversely affected their LVEF. This could have introduced a small degree of variability into the longitudinal analyses. Although our study included 336 women with echocardiographic assessment, this number did not fully meet the originally estimated requirement of 197 participants per group. In addition, the number of women with more severe systolic dysfunction was limited. As a result, small differences between groups may not have been detected, and the confidence intervals should be interpreted with this limitation in mind. Finally, this study was conducted within a single national healthcare system and reflects an earlier treatment era. Although this may limit direct extrapolation to all contemporary patient populations, it represents a clinically relevant group of long-term survivors currently encountered in routine care and provides rare insight into very late cardiac outcomes.

### 4.5 Clinical implications

Currently, no formal international guideline exists for general practitioners regarding the long-term monitoring of systolic function in breast cancer survivors. The recently updated Dutch cardiovascular risk management guideline advises considering optimization of the cardiovascular risk profile in cancer survivors. However, it does not provide specific guidance on the monitoring of cardiac function. In our longitudinal analysis, left ventricular systolic function improved slightly on average over time in both breast cancer survivors and controls with comparable baseline CV risk factors.

The improvement in LVEF was negligible and therefore unlikely to have clinical significance, as it does not alter patient care or management strategies. These findings suggest that, in the absence of additional CV risk factors, routine long-term cardiac monitoring in breast cancer survivors may not be necessary.

## 5 Conclusion

Long-term breast cancer survivors who received chemotherapy with or without radiation therapy exhibit an increased prevalence of mild left ventricular systolic dysfunction. Although breast cancer survivors had a higher odds of mild systolic dysfunction compared with controls, the absolute prevalence of clinically relevant dysfunction was low and most abnormalities were mild. Moreover, LVEF remained stable or slightly improved over time across all groups. Taken together, these findings suggest that, in the absence of additional cardiovascular risk factors, the added value of routine long-term cardiac surveillance beyond standard care may be limited.

## CRediT authorship contribution statement

**L.T. van der Wal:** Writing – review & editing, Writing – original draft, Resources, Project administration, Methodology, Investigation, Formal analysis, Data curation. **S.W.M.C. Maass:** Writing – review & editing, Writing – original draft, Project administration, Investigation, Funding acquisition. **M.R. de Boer:** Writing – review & editing, Validation, Methodology, Funding acquisition, Conceptualization. **M.H. Blanker:** Writing – review & editing, Supervision. **A.P.G. Crijns:** Writing – review & editing, Funding acquisition, Conceptualization. **J.A. Gietema:** Writing – review & editing, Funding acquisition, Conceptualization. **P. van der Meer:** Writing – review & editing, Funding acquisition, Conceptualization. **G.H. de Bock:** Writing – review & editing, Writing – original draft, Supervision, Funding acquisition, Conceptualization. **D. Brandenbarg:** Writing – review & editing, Writing – original draft, Validation, Supervision, Project administration, Methodology, Investigation, Funding acquisition, Data curation, Conceptualization.

## Declaration of generative AI use

During the preparation of this work, the authors used Copilot to assist in generating R codes and to improve readability and language [22]. After using this tool, the authors carefully reviewed and edited the content as needed and took full responsibility for the final version of the published article.

## Funding sources

This work was supported by 10.13039/501100001826ZonMw [grant number 08391052110002].

## Declaration of competing interest

Given her role as editorial board member, G.H. de Bock had no involvement in the peer-review of this article and has no access to information regarding this peer review. The remaining authorsdeclare that they have no known competing financial interests or personal relationships that could have appeared to influence the work reported in this paper.

## Figures and Tables

**Table A.1:** Baseline characteristics at time of breast cancer diagnosis for breast cancer survivors and controls lost to follow-up.

	Breast cancer survivors (N = 184)	Controls (N = 180)
Age breast cancer diagnosis, years, median (IQR)	53.7 (47.8 - 60.0)	52.7 (46.6 - 59.0)
Year of diagnosis, year, median (IQR)	2005.0 (2000.0 - 2007.4)	2003.7 (1999.6 - 2006.8)

Chemotherapy	85 (46.2)	-
Antracycline based chemotherapy	63 (34.2)	-
Cumulative anthracycline dose; mg/m2, median (IQR)^c^	244.4 (228.9 - 300.0)	-
Radiation therapy	162 (88.0)	-
Left sided radiation therapy	79 (42.9)	-
Hormonal therapy	72 (39.1)	1 (0.0)
Trastuzumab	4 (2.2)	-

**Cardiovascular risk factor**^a^	25 (13.6)	24 (13.3)
**Cardiovascular disease**^b^	4 (2.2)	5 (2.8)
**Deceased**	41 (22.3)	20 (11.1)

a Lipid metabolism disorders, hypertension or diabetes mellitus.

b Heart failure, ischemic cardiovascular disease, atrial fibrillation, other cardiac diseases.

c Doxorubicin isotoxic dose, information available for 53 patients.

**Table A.2:** The prevalence of left ventricular dysfunction, diastolic dysfunction and right ventricular dysfunction with a follow-up measurement for breast cancer survivors treated with chemo-with/without radiation therapy and their controls and breast cancer survivors treated with radiation therapy only and their controls.

	Chemotherapy with or without radiation therapy N = 90 (%)	Controls chemotherapy with or without radiation therapy N = 83 (%)	Radiation therapy N = 76 (%)	Controls radiation therapy N = 87 (%)
**Left ventricular dysfunction**				
LVEF <54%	11 (12.2)	2 (2.4)	6 (7.9)	4 (4.6)
LVEF <50%	7 (7.8)	2 (2.4)	3 (3.9)	3 (3.4)
LVEF <45%	1 (1.1)	0 (0.0)	0 (0.0)	0 (0.0)
GLS > −16%	23 (35.4)	9 (13.4)	13 (21.0)	17 (23.9)
**Diastolic dysfunction**				
Diastolic dysfunction guideline 2016	0 (0.0)	1 (1.3)	2 (2.9)	1 (1.2)
Increased LAVI (>34 ml/m^2^)	15 (17.0)	12 (14.5)	19 (25.7)	15 (17.6)
Increased E/e'ratio (>14)	0 (0.0)	2 (2.5)	0 (0.0)	0 (0.0)
**Right ventricular dysfunction**				
Decreased TAPSE (<17 mm)	0 (0.0)	0 (0.0)	1 (1.3)	3 (3.4)
Decreased S' (<9.5 cm/s)	0 (0.0)	0 (0.0)	2 (2.9)	1 (1.2)

GLS (median, IQR)	−17.2 (−18.7 to −15.2)	−18.8 (−20.6 to −17.2)	−17.6 (−19.2 to −16)	−18.6 (−20.0 to −16.8)
E/e' ratio (median, IQR)	6.7 (5.8 - 7.9)	6.4 (5.3 - 7.5)	7.0 (6.2 - 8.2)	7.2 (6.2 - 8.3)
LAVI (median, IQR)	24.7 (21.2 - 31.3)	26.9 (23.7 - 31.0)	27.3 (22.4 - 34.1)	26.4 (23.7 - 30.4)

LVEF: Left ventricular ejection fraction. GLS: Global longitudinal strain. LAVI: Left atrial volume index. TAPSE: tricuspid annular plane systolic excursion.

LVEF available for 90 women treated with chemotherapy with or without radiation therapy (CT), 83 controls CT, 76 women treated with radiation therapy (RT) and 87 controls RT, GLS available for 65 CT, 67 controls CT, 62 RT, 71 controls RT. Diastolic dysfunction available in 76 CT, 78 controls CT, 70 RT and 81 controls RT. LAVI available for 88 CT, 83 controls CT, 74 RT and 85 controls RT. E/e’ available for 83 CT, 80 controls CT, 71 RT and 84 controls RT. TAPSE available for 90 CT, 82 controls CT, 76 RT and 87 controls RT. S′ available for 86 CT, 77 controls CT, 70 RT and 85 controls RT.

**Table A.3:** The prevalence of left ventricular dysfunction, diastolic dysfunction and right ventricular dysfunction at T1 for women with a follow-up measurement, and the comparison between breast cancer survivors and controls.

Echo parameters T1			
	Breast cancer survivors (N = 166)	Controls (N = 170)	
GLS (median, IQR)	−17.8 (−19.9 to −15.8)	−17.6 (−20.2 to −15.8)	
E/e' ratio (median, IQR)	7 (6.1 - 8.4)	7.2 (6.1 - 8.3)	
LAVI (median, IQR)	24.0 (21.0 - 28.0)	24.0 (21.0 - 27.0)	
	N (%)	N (%)	OR (95% CI)

**Left ventricular dysfunction**			
LVEF<54%^a^	27 (16.3)	11 (6.5)	**2.9 (1.4**–**6.0)**
LVEF<50%^a^	8 (4.8)	4 (2.4)	2.1 (0.6 - 7.2)
LVEF<45%^a^	0 (0)	1 (0.6)	-
GLS > −16%	25 (28.7)	30 (27.8)	1.0 (0.6 - 2.0)
**Diastolic dysfunction**			
Diastolic dysfunction guideline 2016	0 (0)	0 (0)	-
Increased LAVI (>34 ml/m^2^)	9 (5.4)	8 (4.7)	1.1 (0.4 - 3.0)
Increased E/e'ratio (>14)	1 (0.6)	2 (1.2)	0.5 (0.0 - 5.8)
**Right ventricular dysfunction**			
Decreased TAPSE (<17 mm)	3 (1.2)	3 (1.8)	0.7 (0.1 - 4.3)
Decreased S' (<9.5 cm/s)	1 (0.7)	3 (1.9)	0.4 (0.4 - 3.4)

LVEF for 166 BC survivors and 161 controls. GLS for 87 BC survivors and 108 controls. Diastolic disfunction for 145 BC survivors and 154 controls. E/e for 159 BC survivors and 167 controls. LAVI for 157 BC survivors and 155 controls. TAPSE for 155 BC survivors and 166 controls. S′ for 149 BC survivors and 159 controls.

a LVEF through eyeballing for 60 BC survivors and 45 controls.
